# Intestinal tumor suppression in *Apc*^Min/+^ mice by prostaglandin D_2_ receptor PTGDR

**DOI:** 10.1002/cam4.251

**Published:** 2014-04-12

**Authors:** Brigette L Tippin, Alan M Kwong, Michael J Inadomi, Oliver J Lee, Jae Man Park, Alicia M Materi, Virgilio S Buslon, Amy M Lin, Lili C Kudo, Stanislav L Karsten, Samuel W French, Shuh Narumiya, Yoshihiro Urade, Eduardo Salido, Henry J Lin

**Affiliations:** 1 Department of Pediatrics, Harbor-UCLA Medical Center and Los Angeles Biomedical Research InstituteTorrance, California; 2 Department of Pathology, Harbor-UCLA Medical Center and Los Angeles Biomedical Research InstituteTorrance, California; 3 NeuroInDx, Inc.Signal Hill, California; 4 Department of Pharmacology, Faculty of Medicine, University of KyotoKyoto, Japan; 5 Department of Molecular Behavioral Biology, Osaka Bioscience InstituteOsaka, Japan; 6 CIBERER, Hospital Universitario CanariasLa Laguna, Spain

**Keywords:** Adenomatous polyposis coli, gastrointestinal neoplasms, PPAR gamma, prostaglandin D_2_ receptor, prostaglandin D_2_ synthases

## Abstract

Our earlier work showed that knockout of hematopoietic prostaglandin D synthase (HPGDS, an enzyme that produces prostaglandin D_2_) caused more adenomas in *Apc*^Min/+^ mice. Conversely, highly expressed transgenic HPGDS allowed fewer tumors. Prostaglandin D_2_ (PGD_2_) binds to the prostaglandin D_2_ receptor known as PTGDR (or DP1). PGD_2_ metabolites bind to peroxisome proliferator-activated receptor *γ* (PPARG). We hypothesized that *Ptgdr* or *Pparg* knockouts may raise numbers of tumors, if these receptors take part in tumor suppression by PGD_2_. To assess, we produced *Apc*^Min/+^ mice with and without *Ptgdr* knockouts (147 mice). In separate experiments, we produced *Apc*^Min/+^ mice expressing transgenic lipocalin-type prostaglandin D synthase (PTGDS), with and without heterozygous *Pparg* knockouts (104 mice). Homozygous *Ptgdr* knockouts raised total numbers of tumors by 30–40% at 6 and 14 weeks. Colon tumors were not affected. Heterozygous *Pparg* knockouts alone did not affect tumor numbers in *Apc*^Min/+^ mice. As mentioned above, our *Pparg* knockout assessment also included mice with highly expressed *PTGDS* transgenes. *Apc*^Min/+^ mice with transgenic PTGDS had fewer large adenomas (63% of control) and lower levels of v-myc avian myelocytomatosis viral oncogene homolog (MYC) mRNA in the colon. Heterozygous *Pparg* knockouts appeared to blunt the tumor-suppressing effect of transgenic PTGDS. However, tumor suppression by PGD_2_ was more clearly mediated by receptor PTGDR in our experiments. The suppression mechanism did not appear to involve changes in microvessel density or slower proliferation of tumor cells. The data support a role for PGD_2_ signals acting through PTGDR in suppression of intestinal tumors.

## Introduction

Prostaglandin studies in intestinal neoplasia usually focus on prostaglandin E_2_ (PGE_2_), a pro-tumorigenic compound 1–4. In an opposite effect, knockout of the gene for hematopoietic prostaglandin D synthase (HPGDS) caused more adenomas in *Apc*^Min/+^ mice. Moreover, high HPGDS production from transgenes allowed fewer 5. Prostaglandin D_2_ (PGD_2_) and PGE_2_ are both made from PGH_2_, so *Hpgds* knockouts could have shunted conversion of PGH_2_ to PGE_2_. Likewise, *HPGDS* transgenes could have drawn prostaglandin synthesis away from PGE_2_.

Lewis lung cancer cells implanted onto the backs of mice lacking the PGD_2_ receptor (PTGDR, also known as DP1), grew faster than tumors implanted onto wild-type mice 6. Furthermore, the PTGDR agonist, BW245C, reduced tumor growth. These results support a role for PGD_2_ itself.

Tumor suppression by PGD_2_ might also occur through inhibition of inflammatory genes by molecules that bind PGD_2_ metabolites. For example, PGD_2_ metabolites bind to peroxisome proliferator-activated receptor *γ* (PPARG). Such binding can induce conjugation of small ubiquitin-related modifier-1 (SUMO-1) to PPARG. SUMOylation is thought to increase PPARG binding to nuclear receptor corepressor complexes, causing transrepression of inflammatory genes 7. Additionally, 15-deoxy-Δ^12,14^-PGJ_2_ may down-regulate inflammatory genes, through covalent binding to nuclear factor-*κ*B or I*κ*B kinase 8.

Here, we show that knockouts of *Ptgdr* increased tumor numbers in *Apc*^Min/+^ mice, indicating that PGD_2_ and PTGDR act to suppress tumors. PPARG had smaller effects in our experiments.

## Material and Methods

### Mice

The protocol and procedures were approved by the Institutional Animal Care and Use Committee (IACUC) at Los Angeles Biomedical Research Institute. C57BL/6, FVB/N, and *Apc*^Min/+^ (C57BL/6; no. 002020) mice came from Jackson (Bar Harbor, ME), as did mice carrying the Cre transgene controlled by the adenovirus EIIa promoter [Tg(EIIa-Cre) C5379Lmgd/J; FVB/N strain; no. 003314] 9. Mice in which exon 2 of the *Pparg* gene is flanked by loxP sites were from F. Gonzalez (*Pparg*^flox/flox^ FVB/N mice).

To produce *Apc*^Min/+^ mice with heterozygous *Ptgdr* knockouts, we crossed male *Apc*^Min/+^ mice with female homozygous *Ptgdr* knockout mice 10. Male *Apc*^Min/+^ mice with heterozygous or homozygous *Ptgdr* knockouts were then bred with female homozygous *Ptgdr* knockout mice to produce *Apc*^Min/+^ mice with homozygous *Ptgdr* knockouts (all 100% C57BL/6).

Our *PTGDS* transgenic mice (line B20; FVB/N) overexpress human PTGDS in all tissues 11. Reported basal brain levels of PGD_2_ were 1.5-fold higher than wild-type levels and rose fivefold upon stimulation. PGE_2_ levels did not change. The mice had more eosinophilia in a bronchial asthma model, compared to *HPGDS* transgenic mice 12.

To generate heterozygotic *Pparg* knockout mice, we crossed *Pparg*^flox/flox^ mice with Tg(EIIa-Cre) mice and identified heterozygotes lacking exon 2 of the *Pparg* gene (*Pparg*^+/−^ mice; all FVB/N) 13. We then crossed female *Pparg*^+/−^ FVB/N mice with male *Apc*^Min/+^ C57BL/6 mice to produce *Apc*^Min/+^ *Pparg*^+/−^, *Apc*^Min/+^, and *Pparg*^+/−^ mice, all on an F_1_ mixed background. Similarly, we bred *PTGDS* transgenic FVB/N males with C57BL/6 females to produce transgenic mice on an F_1_ C57BL/6 × FVB/N background. We intercrossed these various offspring to obtain additional mice with desired genotypes. Fifteen of the 104 mice used were C57BL/6 × FVB/N F_1_ mice, and 89 were from matings of F_1_ mice or mice in later generations (all 50% C57BL/6).

### Intestinal histopathology and definitions of tumor sizes

Adenomas were counted histologically at 6 or 14 weeks, without knowing genotypes 5. We used 24 Swiss roll sections spaced 150 *μ*m apart for *PTGDS* transgenic mice, *Pparg* knockout mice, and their controls. We used 10 Swiss roll sections (250 *μ*m apart) for *Ptgdr* knockout mice and their controls. Tumors sizes were gauged by the number of sections spanned. *Small* tumors were defined as those seen in only 1 section. *Large* tumors were those with profiles in multiple sections. Mitotic figures were identified as described 14.

### Statistical analyses of tumor data

Tumor data were analyzed by nonparametric methods (Kruskal–Wallis and Mann–Whitney), because numbers of tumors per mouse did not follow a Gaussian distribution. We analyzed total, small, large, and colon tumors. We also calculated ratios of the geometric mean number of tumors in genetically modified mice to the geometric mean number in controls. Ratios were estimated from differences in logarithm-transformed tumor numbers. For the colon, we added 0.5 to all numbers of tumors before taking logarithms, to handle zero values. Data from 6- and 14-week-old mice were analyzed separately. These statistical methods were also used to reanalyze tumor data from *Apc*^Min/+^ mice with transgenic HPGDS (and controls) from earlier work 5.

### Immunohistochemistry

Antibodies used were: mouse monoclonal anti-human PTGDS 15; rabbit polyclonal anti-human HPGDS; mouse monoclonal anti-rat proliferating cell nuclear antigen (PCNA); and rat monoclonal anti-mouse CD31. Staining for HPGDS, PCNA, and CD31 was done on slides from *Apc*^Min/+^ mice with transgenic HPGDS from earlier work 5.

### In situ hybridization

Digoxigenin-labeled probes were prepared by in vitro transcription from a linearized plasmid vector containing the mouse PTGDR cDNA (DIG RNA labeling kit; Roche; Indianapolis, IN). T7 RNA polymerase was used to make anti-sense probes. SP6 RNA polymerase was used to prepare control sense probes 16.

### mRNA analyses by reverse transcription and real-time PCR (RT-PCR)

Primers, probes, and procedures for preparing RNA and determining copy numbers of RNA transcripts are in Supporting Information. Assays for v-myc avian myelocytomatosis viral oncogene homolog (MYC), GAPDH, and vascular endothelial growth factor A (VEGFA) were performed with kits (Applied Biosystems; Grand Island, NY; Mm00487803_m1, Mm99999915_g1, Mm00437304_m1, respectively).

## Results

### Tumor scoring

**Figure 1:**
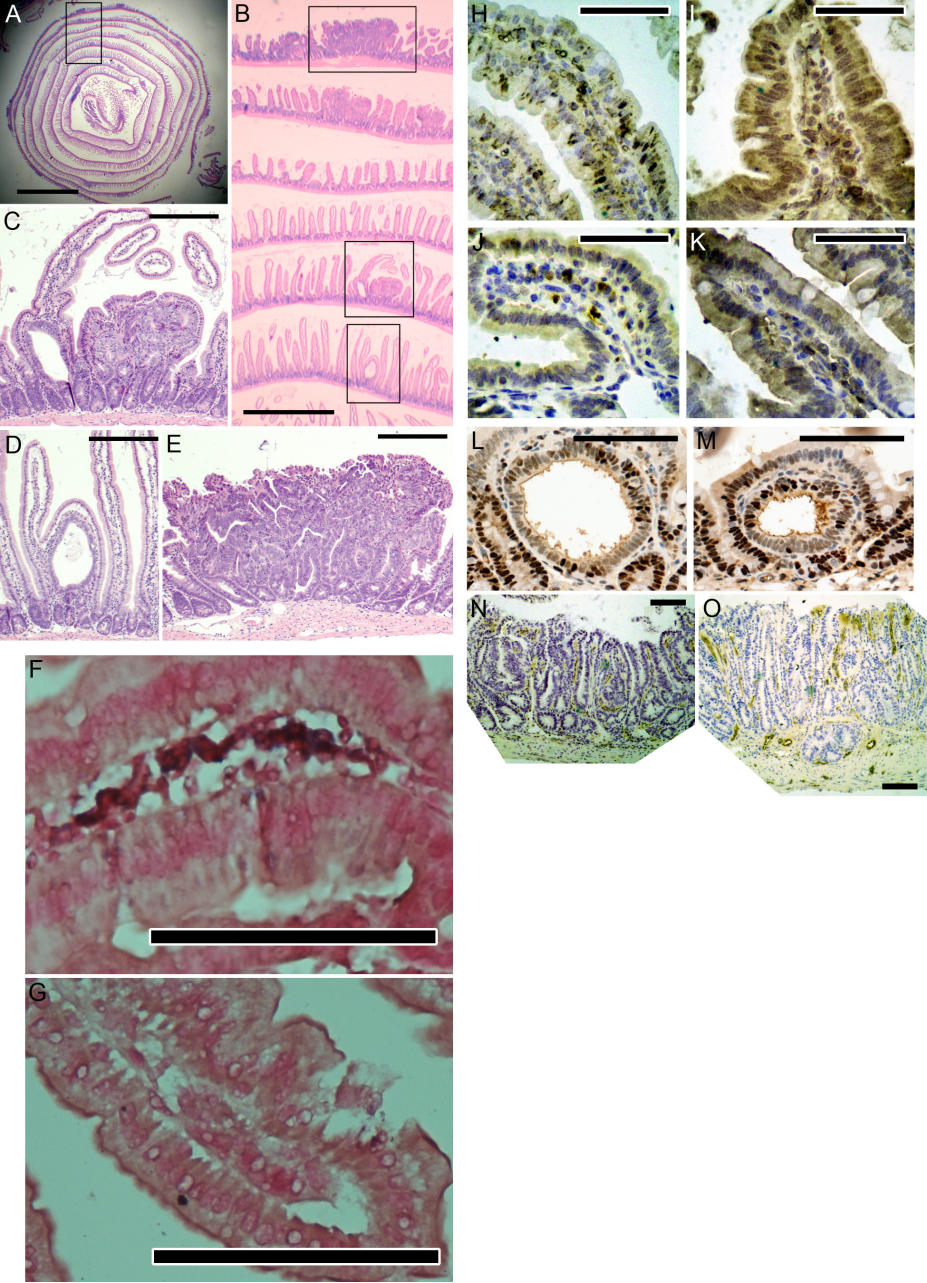
(A–E) Swiss roll section (14 weeks). (A) The box outlines B. Scale bar, 5 mm. (B) The top, middle, and bottom boxes outline E, C, and D, respectively. Scale bar, 1 mm. (C) An early adenoma abutting against a larger adenoma. Scale bar, 200 *μ*m. (D) An early adenoma expanding the villus base. Scale bar, 200 *μ*m. (E) A large adenoma. Scale bar, 200 *μ*m. (F–G) In situ hybridization for PTGDR (12 weeks). (F) Detection of PTGDR mRNA with antisense probes. PTGDR mRNA appears as blue deposits in stromal cells, in a pattern consistent with lymphocytes or monocytes, or both. (G) Sense probes showed no staining (negative control). Counterstained with neutral red. Scale bar, 100 *μ*m. (H–O) Immunohistochemistry (14 weeks). (H–K) High production of human *PTGDS* and *HPGDS* in transgenic mice, shown by immunoperoxidase staining (with rabbit polyclonal anti-human PTGDS [H, I] or HPGDS [J, K] antibodies). Staining (brown) occurred in all cell types of the small bowel and colon (epithelium and stroma). Scale bars, 50 *μ*m. (H) Small bowel villi from a wild-type mouse. Antibody labeling is mostly in the cytosol of some epithelial cells, with occasional stromal cell staining. (I) A small bowel villus from a *PTGDS* transgenic mouse. Antibody binding is heavy throughout the villus, with a cytoplasmic staining pattern. (J) Small bowel villi from a wild-type mouse. HPGDS staining is mainly within the stroma of villi, not epithelial cells. Earlier studies showed these cells to be macrophages and monocytes. (K) Small bowel villi from an *HPGDS* transgenic mouse, showing heavy antibody staining in all cells (as in I). (L, M) Staining for PCNA in intravillar tumors from *Apc*^Min/+^ mice, with and without *HPGDS* transgenes. There was no consistent difference in staining between mice with and without transgenic HPGDS. Scale bars, 100 *μ*m. (L) An intravillar adenoma from an *Apc*^Min/+^ mouse. (M) An intravillar tumor from an *HPGDS* transgenic *Apc*^Min/+^ mouse. (N, O) Staining for microvessels with anti-CD31 antibodies in tumors from *Apc*^Min/+^ mice with and without *HPGDS* transgenes. There was no consistent difference in staining between mice with and without transgenic HPGDS. Scale bars, 100 *μ*m. (N) A tumor from an *Apc*^Min/+^ mouse. (O) A tumor from an *HPGDS* transgenic *Apc*^Min/+^ mouse.

We histologically examined >35,000 tumors in Swiss roll sections (Fig.1A–E), including 9837 tumors from 147 mice in *Ptgdr* knockout experiments, 21,763 tumors from 104 mice in experiments on *PTGDS* transgenic and *Pparg* knockout mice, and 3431 tumors reexamined from 39 *HPGDS* transgenic mice and controls from earlier work 5.

The earliest tumors were uniglandular, intravillar lesions with a simple cystic configuration, or *intravillar* neoplasms, also known as intravillous microadenomas 17,18, dysplastic crypts 19, and cystic crypts (Fig. S1A and S1D) 20,21. More advanced early tumors may have other dysplastic features, such as extension of dysplastic cells (Fig. S1E), multiple lumina (Fig. S1C and G), loss of epithelial cell polarity (Fig. S1G), pseudo-stratification, or crowding (Fig. S1F).

Intravillar tumors progressed by enlarging, forming adjoining cysts (Figs. S1C and S2), or erupting through the villus surface into the bowel lumen (Figs. S1B and S3). Although early tumors arise from crypts 17,22, we found only a few examples of out-pouching of cysts from crypts (Fig. S4). Serial sections from two tumors (75 sections each) showed that early tumors may have no crypt connection (Fig. S5) 21. Examples of early colon tumors are shown in Fig. S6.

### *Ptgdr* knockouts and intestinal tumors

**Figure 2:**
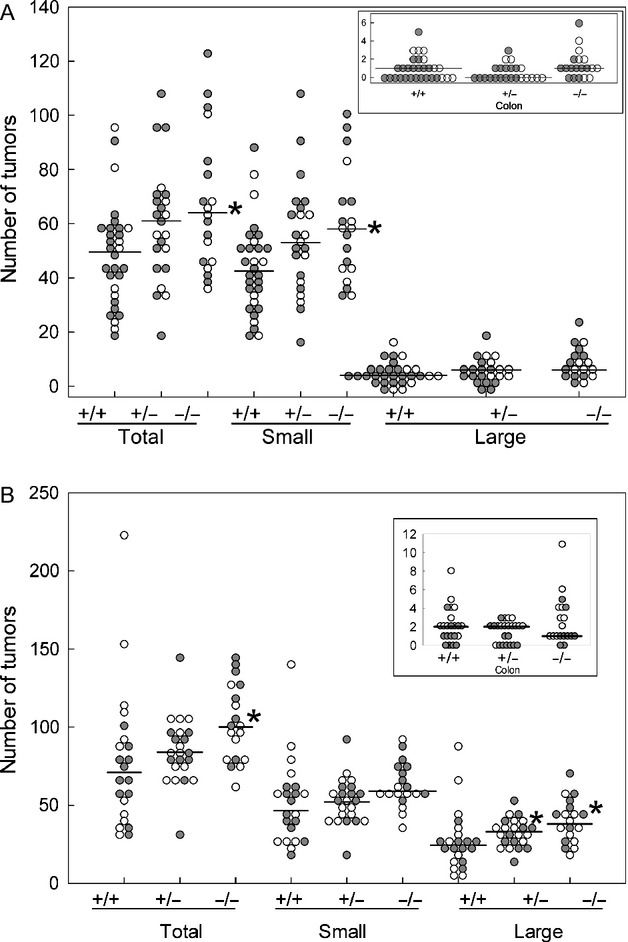
Tumors in *Apc*^Min/+^ mice, with and without *Ptgdr* knockouts (total, small, large, and colon). (A) Tumors at 6 weeks. (B) Tumors at 14 weeks. +/+, control *Apc*^Min/+^ mice. +/− and −/−, *Apc*^Min/+^ mice with heterozygous and homozygous *Ptgdr* knockouts, respectively. Filled symbols: females. Open symbols: males. Horizontal bars: medians. *P <* *0.025. See Tables S1 and S2 for details.

At 6 weeks (Fig.2A), homozygous *Ptgdr* knockouts raised total numbers of tumors (medians 64 vs. 49.5; *P *=* *0.0086; Table S1A) and numbers of small tumors (medians 58 vs. 42.5; *P *=* *0.0089; Table S1B). Large tumors and colon tumors were not affected by *Ptgdr* knockouts at 6 weeks.

At 14 weeks (Fig.2B), heterozygous *Ptgdr* knockouts increased the median number of large tumors (33 vs. 24; *P *=* *0.023; Table S2C). Also at 14 weeks, homozygous *Ptgdr* knockouts raised median numbers of total tumors (100 vs. 71; *P *=* *0.0060; Table S2A) and large tumors (38 vs. 24; *P *=* *0.0040; Table S2C). *Ptgdr* knockouts did not affect small or colon tumors at 14 weeks (Table S2B and D).

To obtain data on occurrence of the earliest tumors, we also scored tumors in ten 3-week-old mice: six *Apc*^Min/+^ mice (3–8 tumors each); three *Apc*^Min/+^ mice with heterozygous *Ptgdr* knockouts (5–11 tumors each); and one *Apc*^Min/+^ mouse with homozygous *Ptgdr* knockouts (11 tumors). However, data from these 10 mice were not included in statistical analyses, because of the age difference.

In situ hybridization for PTGDR mRNA showed consistent, but weak, staining of inflammatory cells in the mucosal stroma (lymphocytes or monocytes, or both; Fig.1F–G). There was no detectable staining in epithelial cells of crypts or villi. Staining with PTGDR antibodies was not conclusive (not shown).

### Expression of transgenic PTGDS

Human *PTGDS* transgenes were highly expressed in the intestines, as measured RT-PCR. Specifically, we found 1.61 × 10^5^ and 8.13 × 10^5^ copies of human *PTGDS* transcripts per nanogram of total RNA in two transgenic mice (geometric mean, 3.6 × 10^5^ copies). These values were comparable to levels for *HPGDS* transgenes in earlier work (7.5 × 10^5^ copies—a 375-fold increase in expression of transgenic *HPGDS* over endogenous mouse *Hpgds*) 5. Immunohistochemistry showed heavy staining of transgenic PTGDS in all intestinal cells (Fig.1H–I). Endogenous mouse PTGDS mRNA was not detectable in the colon.

### Transgenic PTGDS and large tumors

**Figure 3:**
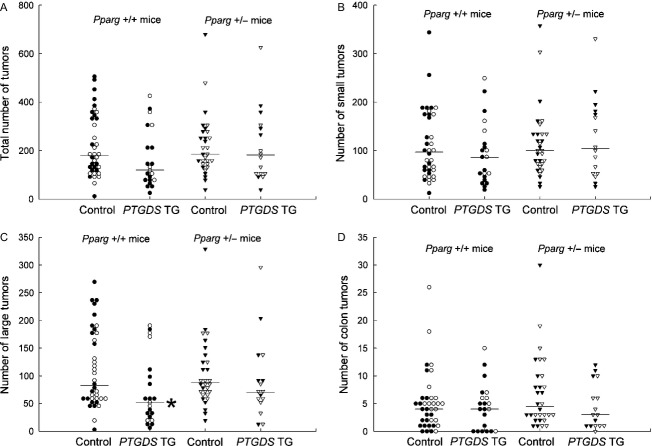
Tumors in *Apc*^Min/+^ mice with *PTGDS* transgenes, with and without heterozygous *Pparg* knockouts (all 14 weeks). Data are for total (A), small (B), large (C), and colon tumors (D). There were statistically significant reductions in numbers of large tumors (C) in *PTGDS* transgenic mice without *Pparg* knockouts. See Table S3 for details. *Pparg* +/+ indicates no *Pparg* knockout. *Pparg* +/− indicates heterozygous *Pparg* knockout. Symbols are as in Figure2. *P <* *0.025.

With 104 *Apc*^Min/+^ mice, we scored intestinal tumors in relation to transgenic PTGDS, with and without heterozygous *Pparg* knockouts. Among mice without *Pparg* knockouts, only large tumors were reduced in number by transgenic PTGDS (medians were 52 vs. 83 for controls; *P *=* *0.011; Fig.3A–D; Table S3). Tumor suppression was also reflected by the ratio of the geometric mean number of large tumors in *PTGDS* transgenic mice to the geometric mean number in controls (ratio = 0.56 for large tumors; 95% confidence interval 0.34–0.92; Table S3C). Large tumors were >150–300 *μ*m in diameter, based on the spacing between sections.

We measured colon mRNA levels for VEGFA and MYC, relative to endogenous GAPDH transcript levels. *PTGDS* transgenes lowered median levels of MYC and VEGFA transcripts by 50% in *Apc*^Min/+^ mice (Fig. S7).

### Heterozygotic *Pparg* knockouts and transgenic PTGDS

Without *PTGDS* transgenes, the numbers of tumors in heterozygotic *Pparg* knockout mice were comparable to numbers in mice without *Pparg* knockouts (Fig.3; Table S3; see “Control”). Thus, heterozygous *Pparg* knockouts alone did not increase tumors in *Apc*^Min/+^ mice.

On the other hand, *Apc*^Min/+^ mice with both transgenic PTGDS and heterozygotic *Pparg* knockouts had intermediate numbers of large tumors. Specifically, going by medians, there were 52 large tumors in mice with *PTGDS* transgenes alone, 88 in mice with heterozygotic *Pparg* knockouts alone, and 70 in mice with both mutations (Table S3C). Similarly, the ratio of the mean number of large tumors in *PTGDS* transgenic mice to the mean number in controls was 0.56 for mice without heterozygotic *Pparg* knockouts (95% confidence interval, 0.34–0.92), compared to 0.78 for mice with heterozygotic *Pparg* knockouts (95% confidence interval, 0.48–1.26).

### PTGDS versus HPGDS

As mentioned above, RT-PCR showed similar expression of transgenic *PTGDS*, compared to transgenic *HPGDS* (as measured in our previous work) 5. Also, immunohistochemistry showed high levels of PTGDS and HPGDS (Fig.1H–K). Both experiments scored tumors in the same way (24 Swiss roll sections; 150 *μ*m between sections). Therefore, we reanalyzed slides from *HPGDS* transgenic mice from our first report 5 to directly compare PTGDS to HPGDS (Fig. S8; Table S4). Ratios of the mean total number of tumors in transgenic mice to the mean total in controls were 0.70 for PTGDS, compared to 0.28 for HPGDS (Tables S3A and S4A). Thus, HPGDS may be two times stronger than PTGDS in suppressing tumors.

We assessed tumor cell proliferation in relation to *HPGDS* transgenes, by use of immunohistochemistry with anti-PCNA antibodies. Again, we used slides from our earlier work on *Apc*^Min/+^ mice with HPGDS transgenes 5. We focused on intravillar tumors, because they are fairly uniform in size. There was no difference in PCNA staining in intravillar tumors in *HPGDS* transgenic versus nontransgenic *Apc*^Min/+^ mice (Fig.1L–M). We also counted mitotic figures in all intravillar tumors of 24 *HPGDS* transgenic mice and 15 *Apc*^Min/+^ controls. There were 0.18 mitoses per tumor in *HPGDS* transgenic *Apc*^Min/+^ mice (33 mitotic figures in 182 intravillar tumors), compared to 0.11 mitoses per tumor in non-transgenic *Apc*^Min/+^ mice (64 mitotic figures in 587 intravillar tumors). Thus, transgenic HPGDS did not reduce tumor cell proliferation.

Immunohistochemistry with anti-CD31 antibodies showed no consistent difference in microvessel staining between *HPGDS* transgenic and nontransgenic tumors (Fig.1N–O). Thus, microvessel growth does not appear to explain occurrence of fewer tumors with PGD_2_.

### Tumors in eight mutants with altered PGD_2_ synthesis or binding

We have now analyzed tumors in eight different *Apc*^Min/+^ mouse mutants that have altered PGD_2_ production or binding, due to knockouts or transgenes. Some experiments used different procedures for cutting sections. For example, we used up to 24 Swiss roll sections for scoring tumors in our first report 5 and in the PTGDS and PPARG experiments shown here. Alternatively, we used 10 sections per Swiss roll in the PTGDR experiments, because reanalysis of earlier data showed that the same conclusions can be reached with 8–10 sections.

**Figure 4:**
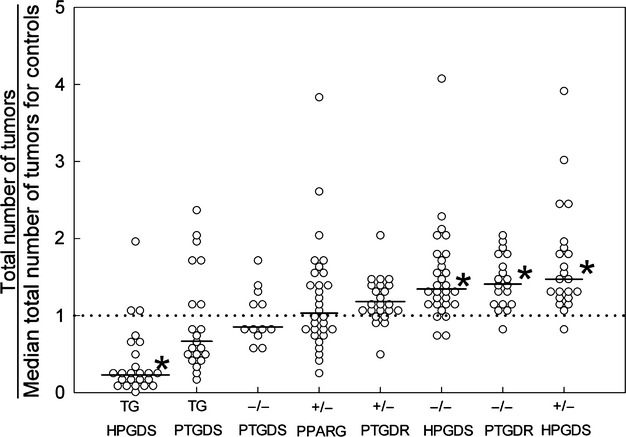
Tumors in *Apc*^Min/+^ mice with various mutations that affect PGD_2_ production or binding (all at 14 weeks). Total numbers of tumors scored for each mouse were divided by the median total number of tumors scored among that mouse's controls. *HPGDS* transgenes are the most tumor-suppressive mutations, whereas homozygous *Ptgdr* knockouts and homozygous and heterozygous *Hpgds* knockouts are the most tumorigenic. The dotted line represents median control values (defined as 1.0). TG, transgenic; +/−, heterozygous knockouts; −/−, homozygous knockouts. Horizontal bars, median values: *HPGDS* TG, 0.23; *PTGDS* TG, 0.67; *Ptgds* −/−, 0.85; *Pparg* +/−, 1.03; *Ptgdr* +/−, 1.18; *Hpgds* −/−, 1.34; *Ptgdr* −/−, 1.40; *Hpgds* +/−, 1.47. *P <* *0.05.

To compare data across experiments, we converted the total number of tumors for each mouse to a “multiple of the median” value. Specifically, we divided the total number of tumors for each mouse by the median number of tumors for that mouse's controls. By this analysis, the most tumor-promoting mutations were *Hpgds* knockouts and homozygous *Ptgdr* knockouts—raising tumor numbers 40% above control values (Fig.4; all mice were analyzed at 14 weeks). In contrast, *HPGDS* transgenes were the most tumor-suppressing mutations—reducing tumor numbers to 20–30% of the control value.

### Female versus male *Apc*^Min/+^ mice

To assess female–male differences in tumor numbers at 14 weeks, we used current data and two earlier reports 5,23 (for a total of 61 female and 75 male *Apc*^Min/+^ mice; Fig. S9). Males and females had similar numbers of intestinal tumors (ratio of tumors in males vs. females, 0.82; *P *=* *0.069). However, males had more colon tumors (ratio of colon tumors in males vs. females, 1.6; *P *=* *0.0002). Results are consistent with McAlpine et al. 24.

## Discussion

### PTGDR and intestinal tumors

Homozygous deletion of the gene for PGD_2_ receptor PTGDR led to 30–40% more intestinal tumors in *Apc*^Min/+^ mice. The result supports an interpretation that PTGDR mediates tumor inhibition by PGD_2_ in these mice. We now have data on eight different *Apc*^Min/+^ mouse mutants, each with a different alteration in PGD_2_ production or binding. Homozygous *Ptgdr* and homozygous or heterozygous *Hpgds* knockout mutations are the most pro-tumorigenic. On the other hand, *HPGDS* transgenes are the most tumor-suppressive mutations—lowering numbers of tumors by 70-80% (Fig.4).

There was no detectable staining of PTGDR mRNA in the epithelium of intestinal crypts or villi. However, PTGDR mRNA was consistently detected in inflammatory cells in the mucosal stroma (Fig.1F–G). Tissue-specific gene knockouts will be needed to more conclusively identify the cells that respond to PGD_2_.

Mutoh et al. 25 treated homozygous *Ptgdr* knockout mice with azoxymethane starting at 7 weeks and examined colons at 12 weeks. They did not find more aberrant crypt foci in the colons of knockout mice, compared to controls. Our results are consistent with Mutoh et al., because we did not observe more colon tumors at 6 or 14 weeks with *Ptgdr* knockouts (Fig.2A–B). However, a role for PTGDR in colon tumor growth is supported by human data. Gustafsson et al. 26 found fivefold lower expression of PTGDR in colorectal cancers, compared to normal tissues (62 tumors and 43 normal tissues, from 62 patients). Galamb et al. 27 showed a trend toward decreased PTGDR expression going from normal tissues, to adenomas, to early cancers, and to advanced cancers.

### Comparison of PTGDS and HPGDS effects

Transgenic PTGDS in *Apc*^Min/+^ mice reduced numbers of large adenomas (>150–300 *μ*m; Fig.3C; Table S3C). In this way, PTGDS had a tumor blocking effect. However, transgenic PTGDS was less effective than transgenic HPGDS in suppressing tumors (Fig.4). Reasons are unknown. A difference between PTGDS and HPGDS is secretion of PTGDS into body fluids, whereas HPGDS stays in the cytosol 28. We recognize that our comparison between PTGDS and HPGDS is based on only one transgenic mouse line for each mutant. But these lines had comparable numbers of PTGDS or HPGDS mRNA transcripts in the intestines (3.6 × 10^5^ and 7.5 × 10^5^ copies, respectively).

Transgenic PTGDS was associated with lower colon expression of MYC (Fig. S7). MYC is a major part of WNT signaling following *Apc* loss 29. Moreover, disruption of *Myc* restores the normal appearance of intestinal crypts in mice with intestine-specific *Apc* knockouts 30. Thus, lower intestinal levels of MYC may be part of the tumor preventive mechanism of PGD_2_.

Reduced levels of VEGFA mRNA were also seen in PTGDS transgenic mice (Fig. S7). The finding is consistent with VEGFA effects in *Apc* mice 31. However, we did not see a decrease in microvessel density in large tumors in *Apc*^Min/+^ mice with transgenic HPGDS (Fig.1N–O). Thus, tumor suppression by PGD_2_ did not appear to involve antiangiogenesis in our experiments 32, at a level detectable by anti-CD31 immunohistochemistry.

Transgenic HPGDS did not reduce PCNA immunostaining in early tumors in *Apc*^Min/+^ mice (Fig.1L–M). PCNA is a marker of intestinal cell proliferation and belongs to the family of sliding DNA clamps that bind factors at replication forks 33. Similarly, transgenic HPGDS did not lower numbers of mitotic figures in early tumors. Thus, PGD_2_ does not appear to suppress tumors by lowering rates of tumor cell division.

A possible explanation is increased tumor cell death with PGD_2_ and PTGDR, as shown by Lewis lung cancer cells implanted onto the backs of mice 6. Alternatively, PGD_2_ may prevent tumors by slowing initiation.

### PPARG and intestinal tumors

Heterozygous *Pparg* knockouts alone did not increase the numbers of tumors in our *Apc*^Min/+^ mice. The result is consistent with earlier reports 24,34. However, McAlpine et al. 24 found ∼30% more tumors in male *Apc*^Min/+^ mice with heterozygous or homozygous intestine-specific *Pparg* deletions.

In our transgenic mice with PTGDS overproduction and reduced adenoma occurrence, the decrease in numbers of large tumors caused by PTGDS appeared blunted in heterozygous *Pparg* knockout mice (Fig.3C and Table S3C). Such blunting could be compatible with tumor suppression by PGD_2_ metabolites bound to PPARG 35, when PGD_2_ production is increased. A limitation in our experiments with heterozygous *Pparg* knockouts and *PTGDS* transgenes was the use of mice with mixed C57BL/6-FVB/N backgrounds (all 50% C57BL/6, but not all F_1_). However, fairly large numbers of mice were used in the *Pparg* experiments (104 in total). The 147 mice in the *Ptgdr* knockout experiments were all 100% C57BL/6.

### PGD_2_ and inflammation

Mechanisms for tumor suppression by PGD_2_ in the intestines have not been proven, but useful information is available. For example, in the skin 36 and lung 37, PGD_2_ delays migration of dendritic cells to draining lymph nodes, where T cells are primed. PGD_2_ also reduces the ability of dendritic cells to stimulate naïve T cells 38,39. In the intestinal mucosa, dendritic cells produce IL-23, to stimulate release of IL-22 by immune cells (innate lymphoid cells 40,41, T_H_17 cells 42, and T_H_22 cells 43). In turn, IL-22 induces proliferation of epithelial cells, production of inflammatory mediators, and release of antimicrobial proteins, to guard against invaders 44. This cytokine can be neutralized by IL-22-binding protein, a soluble receptor also made by dendritic cells in the colon. Huber et al. 45 showed that IL-22 gene knockouts allowed fewer tumors in *Apc*^Min/+^ mice, whereas knockouts of IL-22-binding protein caused more (in the colon). Further work is needed to determine if these functions of dendritic cells explain tumor suppression by PGD_2_. Identification of mechanisms involving PGD_2_ and PTGDR may suggest molecular targets for tumor prevention studies.

## Conclusions

By scoring tumors in *Apc*^Min/+^ mice histologically at 6 and 14 weeks, we showed that homozygous knockouts of the gene for the PGD_2_ receptor, PTGDR, raised median numbers of tumors by 30–40%. The results support an interpretation that PGD_2_ is a tumor-suppressing molecule, acting through PTGDR. Heterozygous *Pparg* knockouts had smaller effects in our experiments. The observation that PGD_2_ and PTGDR can affect tumorigenesis may have impact for prevention.

## Supporting Information

**Figure S1.** Examples of early stage intravillar neoplasms (A and D) and later stage tumors (B, C, E–G) in the small bowel of *Apc*^Min/+^ mice. Arrows indicate single, intravillar, neoplastic glands. (A) An early stage intravillar neoplasm with a single neoplastic gland lying at the level of normal glands and displacing them. (B) Profile of an intravillar neoplasm that progressed by erupting upward through the villus surface and opening into the lumen. Serial sections of this tumor are shown in Figure S3. (C) Profile of an intravillar neoplasm that progressed by forming multiple attached cysts. Serial sections of this lesion are shown in Figure S2. (D) Higher magnification view of an early intravillar neoplasm (not the same tumor as shown in A). (E) A later stage intravillar neoplasm consisting of a single neoplastic gland between the stalk of the villus and underlying normal glands, with extension of neoplastic cells (outlined by asterisks) beyond the gland. (F) A later stage intravillar neoplasm in which the glandular cells display crowding and pseudo-stratification. (G) A more complex, later stage intravillar neoplasm, characterized by polarized neoplastic glandular columnar cells (arrow), multiple neoplastic glandular lumina in a cribriform pattern (two examples are indicated by arrowheads), and solid areas of nonpolarized cells (asterisks). Hematoxylin and eosin staining. Scale bars, 100 *μ*m. **Figure S2.** Sections of an intravillar tumor that contains multiple adjoining cystic structures. The sections indicate that intravillar neoplasms can progress by forming new cysts that abut existing cysts. The slides of this tumor (beginning with the section represented by the top left image and ending with the section represented by the bottom right image) spanned 48 sections (4 *μ*m each). However, only 20 sections are shown here, to save space. Scale bar, 100 *μ*m. **Figure S3.** Sections of an intravillar tumor that erupted through the villus surface. The sections indicate that intravillar neoplasms can progress by expanding toward the top of the villus, erupting through the villus surface, and opening into the bowel lumen. The slides of this tumor (beginning with the section represented by the top left image and ending with the section represented by the bottom right image) spanned 31 sections (4 *μ*m each). However, only 18 sections are shown here, to save space. Scale bar, 100 *μ*m. **Figure S4.** Three examples of intravillar tumors that show a connection to a normal crypt. Scale bar, 100 *μ*m. **Figure S5.** Sections of an intravillar neoplasm in the small bowel of an *Apc*^Min/+^ mouse, showing a uniglandular, intravillar lesion with a simple cystic structure. Although tumors arise from crypt cells, we did not observe a connection between the cystic structure and the crypt for this tumor. Thus, early tumors may become fully enclosed or “sealed off.” All mounted sections containing profiles for this tumor are shown here. Scale bar, 100 *μ*m. **Figure S6.** Examples of colon tumors seen at 6 weeks. Tumors at this age are typically small and lie below the mucosal surface. They would be overlooked without histological examination. The inset in A shows a higher magnification view of the tumor. Scale bar, 100 *μ*m (applies to all panels, except the inset). **Figure S7.** Lower expression of VEGFA and MYC in the colon of *Apc*^Min/+^ mice with *PTGDS* transgenes (TG) and without (WT). mRNA was prepared from colon tissue, and expression levels for VEGFA and MYC were quantitated relative to endogenous mouse GAPDH. Plotted points are averages of triplicate measurements in different mice. VEGFA expression in *PTGDS* transgenic mice was approximately 50% of expression in controls (*P *=* *0.022, Mann–Whitney; *P *=* *0.012, *t*-test). MYC expression was also 50% lower in *PTGDS* transgenic mice (*P *=* *0.041, Mann–Whitney; *P *=* *0.050, *t*-test). Filled symbols: females. Open symbols: males. Horizontal bars show medians. **P *<* *0.05. **Figure S8.** Numbers of adenomas in *Apc*^Min/+^ mice with *HPGDS* transgenes (TG) and without (WT). Transgenic HPGDS was associated with statistically significant reductions in the numbers of tumors in all size categories. See Table S4 for median values, ranges, numbers of mice, *P*-values, and ratios of numbers of tumors in *HPGDS* transgenic mice to numbers in controls. Filled symbols: females. Open symbols: males. Horizontal bars indicate medians. **P *<* *0.05. **Figure S9.** Tumor development in female and male *Apc*^Min/+^ mice at 14 weeks. We combined data from the current experiments with data from two earlier reports 5,23. For each mouse, numbers of tumors (in the entire intestine and in the colon) were normalized to the median number among females in the same experiment. For colon tumors, we added 0.5 to the number of tumors before taking the median. Horizontal bars indicate medians. The dotted horizontal lines indicate 1.0 (which is the median value for females). Males (77 mice) and females (61 mice) tended to have similar numbers of total tumors throughout the intestine (median ratio for males to females = 0.82; *P *=* *0.069; A), but males had roughly 60% more colon tumors, compared to females (median ratio for males to females = 1.6; *P *=* *0.0002; B). **Table S1.** Adenomas at 6 weeks in *Apc*^Min/+^ mice with *Ptgdr* knockouts. **Table S2.** Adenomas at 14 weeks in *Apc*^Min/+^ mice with *Ptgdr* knockouts. **Table S3.** Adenomas in *Apc*^Min/+^ mice with *PTGDS* transgenes, with and without heterozygous *Pparg* knockouts. **Table S4.** Adenomas in *Apc*^Min/+^ mice with *HPGDS* transgenes.
