# Supplementary material for: Intestinal tumor suppression in ApcMin/+ mice by prostaglandin D2 receptor PTGDR
Source: Cancer Med. 2014 Apr 12;3(4):1041–51. doi: 10.1002/cam4.251 (PMC4303173; doi:10.1002/cam4.251)
Supplement: Supplementary file 1 [file cam40003-1041-sd1.doc]

**Supporting Information**

**Methods**

**Mouse husbandry.** Mice were kept on a 12-hour light/dark cycle and fed Purina LabDiet 5001 (for *PTGDS* transgenic mice, *Pparg* knockout mice, and their controls; 5% fat) or the AIN-93G diet with 1995 NRC values (for *Ptgdr* knockout mice and their controls; Dyets, Inc.; Bethlehem, PA). Euthanasia was by CO_2_ asphyxiation or isoflurane sedation followed by cervical dislocation.

**PCR genotyping.** Primers for *Ptgdr* knockouts were (annealing at 62 °C; 1.5 mM MgCl_2_): DP2, GGA TCA TCT GGA TGA AAC ACC (common right primer); DP1, TCG GTC TTT TAT GTG CTC GTG (wild-type left primer; 391 bp product in wild-type mice; 1,500 bp product in knockout mice); and Neo I, CCC GTG ATA TTG CTG AAG AGC (knockout left primer, 340 bp product). Primers for *PTGDS* transgenes were: h-LPGDS f2 Tg, CGA GCT GGC TCC AGG AGA AGA AGG C; and m-LPGDS r, GCT GAG AGG GTG GCC ATG CGG AAG (annealing at 71.5 °C; 2.0 mM MgCl_2_; 300 bp product). DNA templates were from dried blood on Whatman 903 paper (GE Healthcare; Piscataway, NJ). Genotyping for *Pparγ*^(13)^ and *Apc*^Min(5)^ was as described.

**Collection of intestines.** Mice were euthanized at 6 or 14 weeks. Intestines were immediately removed, placed in ice-cold Ringer solution (9 g NaCl, 300 mg KCl, 350 mg CaCl_2_ dihydrate in 1 liter of deionized water), opened longitudinally (without flushing), fixed in formalin, embedded in paraffin as Swiss rolls (inside out), sectioned (4 μm), mounted on slides, and stained with hematoxylin and eosin.

**Immunohistochemistry.** Antibodies were: mouse monoclonal anti-human PTGDS (13.7 mg/ml); rabbit polyclonal anti-human HPGDS (Cayman Chemical; Ann Arbor, MI); mouse monoclonal anti-rat proliferating cell nuclear antigen (PCNA; BioGenex; San Ramon, CA); rat monoclonal anti-mouse CD31 (Abcam; Cambridge, MA); rabbit polyclonal anti-human CD3 (Abcam); and rabbit monoclonal anti-human CD11b (Abcam).

For PTGDS staining, paraffin-embedded sections were de-waxed and hydrated. Antigen retrieval was in 10 mM Tris-HCl, pH 9.5, at 80 °C for 30 min. Tissue was next treated with 0.1% Triton X-100 in phosphate buffered saline (PBS) for 10 min, and then incubated overnight with the anti-PTGDS antibody (diluted 1:5,000 in 3% bovine serum albumin-PBS). After blocking endogenous peroxidase with 3% H_2_O_2_-methanol solution (15 min) and 3 washes in PBS (5 min each), slides were treated with horseradish peroxidase-conjugated anti-rabbit serum (30 min; Dako; Glostrup, Denmark).  After 3 more PBS washes, a DAB-H_2_O_2_ solution was used as chromogen, and some sections were counterstained with hematoxylin.

HPGDS staining was as described (5; dilution 1:1,500).

For proliferating cell nuclear antigen (PCNA), we used slides with sections that had been previously stained with hematoxylin and eosin. Cover slips were removed by soaking in xylol for >4 days. Sections were washed with absolute, 95% and 85% alcohol, and tap water. After treatment with 3% H_2_O_2_-methanol solution, then sodium 1 mM EDTA buffer (pH 8.0; 3 min in a pressure cooker), and then 0.1% Tween 20 in 20 mM Tris-HCl buffer (pH 7.2-7.4), sections were incubated with the antibody at room temperature for 1 hour (diluted 1:4,000). We detected antigens by the use of a biotinylated secondary antibody and streptavidin-horseradish peroxidase labeling (Cell Marque; Rocklin, CA), with diaminobenzidine as the chromagen.

For CD31, deparaffinized sections were treated with 10 μg/ml proteinase K (Roche; Mannheim, Germany) in PBS for 15 min at room temperature, followed by 10 min in 0.1% Triton X-100 in PBS. Next, we incubated sections with the antibody in 3% BSA-PBS overnight (diluted 1:50-1:100).  After blocking endogenous peroxidases with 0.3% H_2_O_2_ in methanol for 15 min, signal detection was with biotinylated anti-rat IgG (Vector Laboratories; Burlingame, CA), followed by avidin-biotin-peroxidase complexes and diaminobenzidine as the chromogen.

***In situ* hybridization.** From a mouse PTGDR cDNA (clone IRCLp5011G1014D, Source Bioscience; Nottingham, U.K.), we subcloned a 1.9 kb EcoRI-HindIII restriction fragment containing the coding sequence into vector pGEM4Z (Promega; Madison, WI). Digoxigenin-labeled probes were prepared by *in vitro* transcription from linearized vector (DIG RNA labeling kit; Roche). T7 RNA polymerase was used to make anti-sense probes, and SP6 RNA polymerase was used to make control sense probes.

For hybridization, 8 μm paraffin-embedded sections were mounted on aminoalkylsilane-coated slides, to prevent loss of tissue sections during the extensive treatments of the hybridization procedure. Proteolytic digestion for 15 min at 37 °C with proteinase K (20 μg/ml in PBS) was followed by a 5 min rinse in 0.2% glycine in PBS and 2 rinses of 5 min in PBS. Sections were then re-fixed for 15 min in 4% formaldehyde (in PBS) and washed twice in PBS for 5 min.

Sections were pre-hybridized in hybridization mix (50% formamide, 5X SSC, 40 μg/ml salmon sperm DNA) without probe for 1 hr at 70 °C and then hybridized overnight at 70 °C. The probe concentration was ~1 ng/μl. After hybridization, sections were rinsed in 2X SSC, washed for 1 hr at 65 °C in 2X SSC, and then washed at high stringency in 0.1X SSC at 65 °C for 1 hr. After a 5-min wash in TN (100 mM Tris-HCl pH 7.5, 150 mM NaCl), probe bound to the section was immunologically detected by use of a sheep anti-digoxigenin Fab fragment covalently coupled to alkaline phosphatase. Nitro-blue tetrazolium and 5-bromo-4-chloro-3'-indolyphosphate (NBT/BCIP) was the chromogenic substrate, essentially according to the manufacturer's protocol (Roche). Briefly, a 1:5000 dilution of anti-digoxigenin antibody was incubated for 2 hr, washed with TN twice for 15 min each, equilibrated with alkaline TNM (100 mM TrisHCl pH 9.5, 150 mM NaCl, 50 mM MgCl_2_), and developed with NBT/BCIP solution in TNM for 30 min. Counterstaining was with neutral red. Sections were then rinsed in water, dehydrated, and mounted.

**mRNA analyses by reverse transcription and real time PCR (RT-PCR).** Colon specimens (100 mg) were placed in RNAlater (Ambion; Austin, TX) and frozen at the time of sacrifice. RNA was extracted, purified (RNeasy Lipid Tissue Mini Kit, Qiagen; Germantown, MD), reverse transcribed to cDNA, and quantitated via RT-PCR.

To quantitate intestinal expression of the human *PTGDS* transgene, oligonucleotide primers and probes were designed by Primer Express software (Applied Biosystems; Foster City, CA). Primers for RT-PCR of human *PTGDS* transcripts were: H-lpgds159-F2, AGA AGA AGG CGG CGT TGT C (exon 2) and H-lpgds356-R2, ATG GTT CGG GTC TCA CAC T (exon 3). The fluorogenic probe for human *PTGDS* was 6FAM ATG TGC AAG TCT GTG GTG GCC CC TAMRA (exon 2). Primers for RT-PCR of endogenous mouse *Ptgds* transcripts were: LPGDS-FOR, CCA ACC GGA TAA GTG CAT TCA (exons 5-6) and LPGDS-REV, AGG CCA GGT CAC CAT GTG A (exons 6-7). The probe for mouse *Ptgds* was 6FAM TAA ACG CAG GTG AGA GAA GTC AGT CAG AGG G TAMRA (exon 6).

We estimated copy numbers of transgenic human and endogenous mouse *PTGDS* transcripts by the use of standard curves plotted from known amounts of PCR products encoding human or mouse *PTGDS* cDNA sequences. Specifically, a 493 bp PCR product for human *PTGDS* was amplified from cDNA from transgenic mouse tissues by the use of primers Hlpgds-F1 (TGC AGG AGA ATG GCT ACT CA) and Hlpgds-R1 (TCT CCT TTA ACT CAG CCC TG). Similarly, a 208 bp product for endogenous mouse *Ptgds* was amplified from mouse brain cDNA (Ambion/Life Technologies First Choice PCR-ready cDNA; Grand Island, NY) by the use of primers mLpgds-F2 (TCT ACA CGA GAA CCC AGA CT, exons 4-5) and mLpgds-R2 (CTT GAG AGT GAC AGA GCA AG, exon 7).

**Table S1.** Adenomas at 6 weeks in *Apc*Min/+ mice with *Ptgdr* knockouts†

| *A. Total number of adenomas in the entire intestine* | | |  |  |
| --- | --- | --- | --- | --- |
|  |  |  |  |  |
|  | *Ptgdr* knockout | |  | Control |
|  | +/- | -/- |  | +/+ |
| Median | 61 | 64 |  | 49.5 |
| Range | 18-109 | 37-124 |  | 18-95 |
| Number of mice | 23 | 19 |  | 30 |
| *P*-value^‡^ | 0.024 | 0.0086 |  |  |
| Ratio (95% CI)^§^ | 1.25 (1.00 – 1.57) | 1.42 (1.12 – 1.79) |  |  |
|  |  |  |  |  |
| *B. Number of small*^¶^ *adenomas in the entire intestine* | | |  |  |
|  |  |  |  |  |
|  | *Ptgdr* knockout | |  | Control |
|  | +/- | -/- |  | +/+ |
| Median | 53 | 58 |  | 42.5 |
| Range | 15-109 | 33-101 |  | 18-87 |
| Number of mice | 23 | 19 |  | 30 |
| *P*-value^‡^ | 0.036 | 0.0089 |  |  |
| Ratio (95% CI)^§^ | 1.25 (0.99-1.57) | 1.38 (1.11-1.73) |  |  |
|  |  |  |  |  |
| *C. Number of large*^¶^ *adenomas in the entire intestine* | | |  |  |
|  |  |  |  |  |
|  | *Ptgdr* knockout | |  | Control |
|  | +/- | -/- |  | +/+ |
| Median | 6 | 6 |  | 4 |
| Range | 0-18 | 1-23 |  | 0-17 |
| Number of mice | 23 | 19 |  | 30 |
| *P*-value^‡^ | 0.59 | 0.026 |  |  |
| Ratio (95% CI)^§^ | 1.11 (0.67-1.83) | 1.78 (1.09-2.88) |  |  |
|  |  |  |  |  |
| *D. Total number of adenomas in the colon* | | |  |  |
|  |  |  |  |  |
|  | *Ptgdr* knockout | |  | Control |
|  | +/- | -/- |  | +/+ |
| Median | 0 | 1 |  | 1 |
| Range | 0-3 | 0-6 |  | 0-5 |
| Number of mice | 23 | 19 |  | 30 |
| *P*-value^‡^ | 0.29 | 0.24 |  |  |
| Ratio (95% CI)^§^ | 0.76 (0.51-1.17) | 1.36 (0.85-2.16) |  |  |

^†^ We counted adenomas histologically at 6 weeks of age in 10 Swiss roll sections spaced 250 µm apart. *Ptgdr* knockout indicates mice carrying heterozygous (+/-) or homozygous (-/-) *Ptgdr* knockout mutations. Control indicates *Apc*^Min/+^ mice without *Ptgdr* knockouts (+/+). Data are plotted in Fig. 2A.

^‡^ The *P*-values shown are uncorrected and refer to comparison between the indicated genotype and controls, by use of the Mann-Whitney test. Statistical significance using the Bonferroni correction is achieved if the *P*-value is less than 0.05 divided by 2 (i.e., the number of comparisons among the 3 groups).

^§^ Ratio of the geometric mean number of adenomas in *Ptgdr* knockout mice to the geometric mean number of adenomas in controls, and 95% confidence intervals. Ratios were estimated from differences in logarithm-transformed tumor numbers. For the colon, we added 0.5 to all numbers of tumors before taking the logarithm, in order to handle zero values.

^¶^ Small adenomas were defined as those seen in only 1 section, whereas large adenomas were those with profiles seen in multiple sections (i.e., adenomas >250-500 μm in diameter).

**Table S2.** Adenomas at 14 weeks in *Apc*^Min/+^ mice with *Ptgdr* knockouts^†^

| *A. Total number of adenomas in the entire intestine* | | |  |  |
| --- | --- | --- | --- | --- |
|  |  |  |  |  |
|  | *Ptgdr* knockout | |  | Control |
|  | +/- | -/- |  | +/+ |
| Median | 84 | 100 |  | 71 |
| Range | 33-145 | 61-145 |  | 31-225 |
| Number of mice | 23 | 19 |  | 22 |
| *P*-value^‡^ | 0.16 | 0.0060 |  |  |
| Ratio (95% CI)^§^ | 1.20 (0.93-1.54) | 1.46 (1.12-1.90) |  |  |
|  |  |  |  |  |
| *B. Number of small*^¶^ *adenomas in the entire intestine* | | |  |  |
|  |  |  |  |  |
|  | *Ptgdr* knockout | |  | Control |
|  | +/- | -/- |  | +/+ |
| Median | 52 | 59 |  | 46 |
| Range | 20-93 | 36-90 |  | 19-139 |
| Number of mice | 23 | 19 |  | 22 |
| *P*-value^‡^ | 0.47 | 0.039 |  |  |
| Ratio (95% CI)^§^ | 1.10 (0.87-1.40) | 1.33 (1.04-1.71) |  |  |
|  |  |  |  |  |
| *C. Number of large*^¶^ *adenomas in the entire intestine* | | |  |  |
|  |  |  |  |  |
|  | *Ptgdr* knockout | |  | Control |
|  | +/- | -/- |  | +/+ |
| Median | 33 | 38 |  | 24 |
| Range | 21-71 | 18-71 |  | 7-87 |
| Number of mice | 23 | 19 |  | 22 |
| *P*-value^‡^ | 0.023 | 0.0040 |  |  |
| Ratio (95% CI)^§^ | 1.42 (1.04-1.92) | 1.71 (1.21-2.42) |  |  |
|  |  |  |  |  |
| *D. Total number of adenomas in the colon* | | |  |  |
|  |  |  |  |  |
|  | *Ptgdr* knockout | |  | Control |
|  | +/- | -/- |  | +/+ |
| Median | 2 | 1 |  | 2 |
| Range | 0-3 | 0-11 |  | 0-8 |
| Number of mice | 23 | 19 |  | 22 |
| *P*-value^‡^ | 0.43 | 0.77 |  |  |
| Ratio (95% CI)^§^ | 0.78 (0.48-1.26) | 1.15 (0.68-1.93) |  |  |

^†^ We counted adenomas histologically at 6 weeks of age in 10 Swiss roll sections spaced 250 µm apart. *Ptgdr* knockout indicates mice carrying heterozygous (+/-) or homozygous (-/-) *Ptgdr* knockout mutations. Control indicates *Apc*^Min/+^ mice without *Ptgdr* knockouts (+/+). Data are plotted in Fig. 2B.

^‡^ The *P*-values shown are uncorrected and refer to comparison between the indicated genotype and controls, by use of the Mann-Whitney test. Statistical significance using the Bonferroni correction is achieved if the *P*-value is less than 0.05 divided by 2 (i.e., the number of comparisons among the 3 groups).

^§^ Ratio of the geometric mean number of adenomas in *Ptgdr* knockout mice to the geometric mean number of adenomas in controls, and 95% confidence intervals. Ratios were estimated from differences in logarithm-transformed tumor numbers. For the colon, we added 0.5 to all numbers of tumors before taking the logarithm, in order to handle zero values.

^¶^ Small adenomas were defined as those seen in only 1 section, whereas large adenomas were those with profiles seen in multiple sections (i.e., adenomas >250-500 μm in diameter).

**Table S3.** Adenomas in *Apc*^Min/+^ mice with *PTGDS* transgenes, with and without heterozygous *Pparg* knockouts^†^

| *A. Total number of adenomas in the entire intestine (P = 0.18*^‡^*)* | | | | | | | | |
| --- | --- | --- | --- | --- | --- | --- | --- | --- |
|  | | | | | | | | |
|  |  | Mice without *Pparg* KO | | |  | Mice with *Pparg* KO | | |
|  |  | *PTGDS* TG |  | Control |  | *PTGDS* TG |  | Control |
| Median | | 120 |  | 180 |  | 182 |  | 186 |
| Range | | 29-424 |  | 19-500 |  | 34-628 |  | 43-686 |
| Number of mice | | 21 |  | 37 |  | 16 |  | 30 |
| *P*-value^§^ | |  | 0.041 |  |  |  | 0.70 |  |
| Ratio (95% CI)^¶^ | | 0.70 (0.47-1.04) | | |  | 0.91 (0.60-1.40) | | |
|  |  |  |  |  |  |  |  |  |
| *B. Number of small*^††^ *adenomas in the entire intestine (P = 0.53*^‡^*)* | | | | | | | | |
|  | | | | | | | | |
|  |  | Mice without *Pparg* KO | | |  | Mice with *Pparg* KO | | |
|  |  | *PTGDS* TG |  | Control |  | *PTGDS* TG |  | Control |
| Median | | 86 |  | 97 |  | 104 |  | 101 |
| Range | | 21-247 |  | 16-342 |  | 23-331 |  | 23-354 |
| Number of mice | | 21 |  | 37 |  | 16 |  | 30 |
| *P*-value^§^ | |  | 0.28 |  |  |  | 0.84 |  |
| Ratio (95% CI)^¶^ | | 0.82 (0.57-1.19) | | |  | 0.99 (0.67-1.48) | | |
|  |  |  |  |  |  |  |  |  |
| *C. Number of large*^††^ *adenomas in the entire intestine (P = 0.050*^‡^*)* | | | | | | | | |
|  | | | | | | | | |
|  |  | Mice without *Pparg* KO | | |  | Mice with *Pparg* KO | | |
|  |  | *PTGDS* TG |  | Control |  | *PTGDS* TG |  | Control |
| Median | | 52 |  | 83 |  | 70 |  | 88 |
| Range | | 8-190 |  | 3-267 |  | 11-297 |  | 20-332 |
| Number of mice | | 21 |  | 37 |  | 16 |  | 30 |
| *P*-value^§^ | |  | 0.011 |  |  |  | 0.25 |  |
| Ratio (95% CI)^¶^ | | 0.56 (0.34-0.92) | | |  | 0.78 (0.48-1.26) | | |
|  |  |  |  |  |  |  |  |  |
| *D. Total number of adenomas in the colon (P = 0.45*^‡^*)* | | | | | | | | |
|  | | | | | | | | |
|  |  | Mice without *Pparg* KO | | |  | Mice with *Pparg* KO | | |
|  |  | *PTGDS* TG |  | Control |  | *PTGDS* TG |  | Control |
| Median | | 4 |  | 4 |  | 3 |  | 4 |
| Range | | 0-15 |  | 0-26 |  | 0-12 |  | 1-30 |
| Number of mice | | 21 |  | 37 |  | 16 |  | 30 |
| *P*-value^§^ | |  | 0.73 |  |  |  | 0.16 |  |
| Ratio (95% CI)^¶^ | | 0.77 (0.41-1.47) | | |  | 0.64 (0.36-1.15) | | |

^†^ We counted adenomas histologically at 14 weeks of age in 24 Swiss roll sections spaced 150 µm apart. *PTGDS* TG indicates mice carrying a *PTGDS* transgene. *Pparg* KO indicates heterozygosity for a *Pparg* knockout mutation. Data for parts A-D are plotted in Fig. 3.

^‡^ The *P-*value refers to comparison among all 4 groups by use of the Kruskal-Wallis test.

^§^ The *P*-values shown are uncorrected and refer to comparison between the indicated genotypes by use of the Mann-Whitney test. Statistical significance using the Bonferroni correction is achieved if the *P*-value is less than 0.05 divided by 2 (i.e., the number of comparisons among the 4 groups).

^¶^ Ratio of the geometric mean number of adenomas in *PTGDS* transgenic mice to the geometric mean number of adenomas in controls, and 95% confidence intervals. Ratios were estimated from differences in logarithm-transformed tumor numbers. For the colon, we added 0.5 to all numbers of tumors before taking the logarithm, in order to handle zero values.

^††^ Small adenomas were defined as those seen in only 1 section, whereas large adenomas were those with profiles seen in multiple sections (i.e., adenomas >150-300 μm in diameter).

**Table S4.** Adenomas in *Apc*^Min/+^ mice with *HPGDS* transgenes^†^

| *A. Total number of adenomas in the entire intestine* | | | |
| --- | --- | --- | --- |
|  |  |  |  |
|  | *HPGDS* TG |  | Control |
| Median | 29.5 |  | 128 |
| Range | 6-251 |  | 47-425 |
| Number of mice | 24 |  | 15 |
| *P*-value^‡^ |  | 0.0002 |  |
| Ratio (95% CI)^§^ | 0.28 (0.17-0.47) | | |
|  |  |  |  |
| *B. Number of small*^¶^ *adenomas in the entire intestine* | | | |
|  |  |  |  |
|  | *HPGDS* TG |  | Control |
| Median | 11.5 |  | 58 |
| Range | 4-100 |  | 30-274 |
| Number of mice | 24 |  | 15 |
| *P*-value^‡^ |  | 0.0001 |  |
| Ratio (95% CI)^§^ | 0.23 (0.13-0.40) | | |
|  |  |  |  |
| *C. Number of large*^¶^ *adenomas in the entire intestine* | | | |
|  |  |  |  |
|  | *HPGDS* TG |  | Control |
| Median | 16 |  | 54 |
| Range | 1-151 |  | 16-151 |
| Number of mice | 24 |  | 15 |
| *P*-value^‡^ |  | 0.0015 |  |
| Ratio (95% CI)^§^ | 0.33 (0.18-0.59) | | |
|  |  |  |  |
| *D. Total number of adenomas in the colon* | | | |
|  |  |  |  |
|  | *HPGDS* TG |  | Control |
| Median | 2 |  | 6 |
| Range | 0-7 |  | 1-26 |
| Number of mice | 24 |  | 15 |
| *P*-value^‡^ |  | 0.0001 |  |
| Ratio (95% CI)^§^ | 0.31 (0.18-0.51) | | |

^†^ Data are from slides from mice described in Park et al. (5). *HPGDS* TG indicates *Apc*^Min/+^ mice carrying an *HPGDS* transgene. We counted adenomas histologically at 14 weeks of age in 24 Swiss roll sections spaced 150 µm apart. Data for parts A-D are plotted in Fig. S8.

^‡^ The *P*-values refer to comparison between the indicated genotypes by use of the Mann-Whitney test.

^§^ Ratio of the geometric mean number of adenomas in *HPGDS* transgenic mice to the geometric mean number of adenomas in controls, and 95% confidence intervals. The ratios were estimated from differences in logarithm-transformed tumor numbers. For the colon, we added 0.5 to all numbers of tumors before taking the logarithm, in order to handle zero values.

^¶^ Small adenomas were defined as those seen in only 1 section, whereas large adenomas were those with profiles seen in multiple sections (i.e., adenomas >150-300 μm in diameter).

**Legends to Supplemental Figures**

**Fig. S1.** Examples of early stage intravillar neoplasms (A and D) and later stage tumors (B, C, E, F, and G) in the small bowel of *Apc*^Min/+^ mice. Arrows indicate single, intravillar, neoplastic glands. *A*. An early stage intravillar neoplasm with a single neoplastic gland lying at the level of normal glands and displacing them. *B*. Profile of an intravillar neoplasm that progressed by erupting upward through the villus surface and opening into the lumen. Serial sections of this tumor are shown in Fig. S3. *C*. Profile of an intravillar neoplasm that progressed by forming multiple attached cysts. Serial sections of this lesion are shown in Fig. S2. *D*. Higher magnification view of an early intravillar neoplasm (not the same tumor as shown in panel A). *E*. A later stage intravillar neoplasm consisting of a single neoplastic gland between the stalk of the villus and underlying normal glands, with extension of neoplastic cells (outlined by asterisks) beyond the gland. *F*. A later stage intravillar neoplasm in which the glandular cells display crowding and pseudo-stratification. *G*. A more complex, later stage intravillar neoplasm, characterized by polarized neoplastic glandular columnar cells (arrow), multiple neoplastic glandular lumina in a cribriform pattern (2 examples are indicated by arrowheads), and solid areas of nonpolarized cells (asterisks). Hematoxylin and eosin staining. Scale bars, 100 μm.

**Fig. S2.** Sections of an intravillar tumor that contains multiple adjoining cystic structures. The sections indicate that intravillar neoplasms can progress by forming new cysts that abut existing cysts. The slides of this tumor (beginning with the section represented by the top left image and ending with the section represented by the bottom right image) spanned 48 sections (4 μm each). However, only 20 sections are shown here, to save space. Scale bar, 100 μm.

**Fig. S3.** Sections of an intravillar tumor that erupted through the villus surface. The sections indicate that intravillar neoplasms can progress by expanding toward the top of the villus, erupting through the villus surface, and opening into the bowel lumen. The slides of this tumor (beginning with the section represented by the top left image and ending with the section represented by the bottom right image) spanned 31 sections (4 μm each). However, only 18 sections are shown here, to save space. Scale bar, 100 μm.

**Fig. S4.** Three examples of intravillar tumors that show a connection to a normal crypt. Scale bar, 100 μm.

**Fig. S5.** Sections of an intravillar neoplasm in the small bowel of an *Apc*^Min/+^ mouse, showing a uniglandular, intravillar lesion with a simple cystic structure. Although tumors arise from crypt cells, we did not observe a connection between the cystic structure and the crypt for this tumor. Thus, early tumors may become fully enclosed or “sealed off.” All mounted sections containing profiles for this tumor are shown here. Scale bar, 100 μm.

**Fig. S6.** Examples of colon tumors seen at 6 weeks. Tumors at this age are typically small and lie below the mucosal surface. They would be overlooked without histological examination. The inset in panel A shows a higher magnification view of the tumor. Scale bar, 100 μm (applies to all panels, except the inset).

**Fig. S7.** Lower expression of VEGFA and MYC in the colon of *Apc*^Min/+^ mice with *PTGDS* transgenes (TG) and without (WT). mRNA was prepared from colon tissue, and expression levels for VEGFA and MYC were quantitated relative to endogenous mouse GADPDH. Plotted points are averages of triplicate measurements in different mice. VEGFA expression in *PTGDS* transgenic mice was approximately 50% of expression in controls (*P* = 0.022, Mann-Whitney; *P* = 0.012, *t*-test). MYC expression was also 50% lower in *PTGDS* transgenic mice (*P* = 0.041, Mann-Whitney; *P* = 0.050, *t*-test). Filled symbols, females. Open symbols, males. Horizontal bars show medians. **P* < 0.05.

**Fig. S8.** Numbers of adenomas in *Apc*^Min/+^ mice with *HPGDS* transgenes (TG) and without (WT). Transgenic HPGDS was associated with statistically significant reductions in the numbers of tumors in all size categories. See Table S4 for median values, ranges, numbers of mice, *P*-values, and ratios of numbers of tumors in *HPGDS* transgenic mice to numbers in controls. Filled symbols, females. Open symbols, males. Horizontal bars indicate medians. **P* < 0.05.

**Fig. S9.** Tumor development in female and male *Apc*^Min/+^ mice at 14 weeks. We combined data from the current experiments with data from 2 earlier reports.^(5, 23)^ For each mouse, numbers of tumors (in the entire intestine and in the colon) were normalized to the median number among females in the same experiment. For colon tumors, we added 0.5 to the number of tumors before taking the median. Horizontal bars indicate medians. The dotted horizontal lines indicate 1.0 (which is the median value for females). Males (77 mice) and females (61 mice) tended to have similar numbers of total tumors throughout the intestine (median ratio for males to females = 0.82; *P* = 0.069; panel *A*), but males had roughly 60% more colon tumors, compared to females (median ratio for males to females = 1.6; *P* = 0.0002; panel *B*).

**
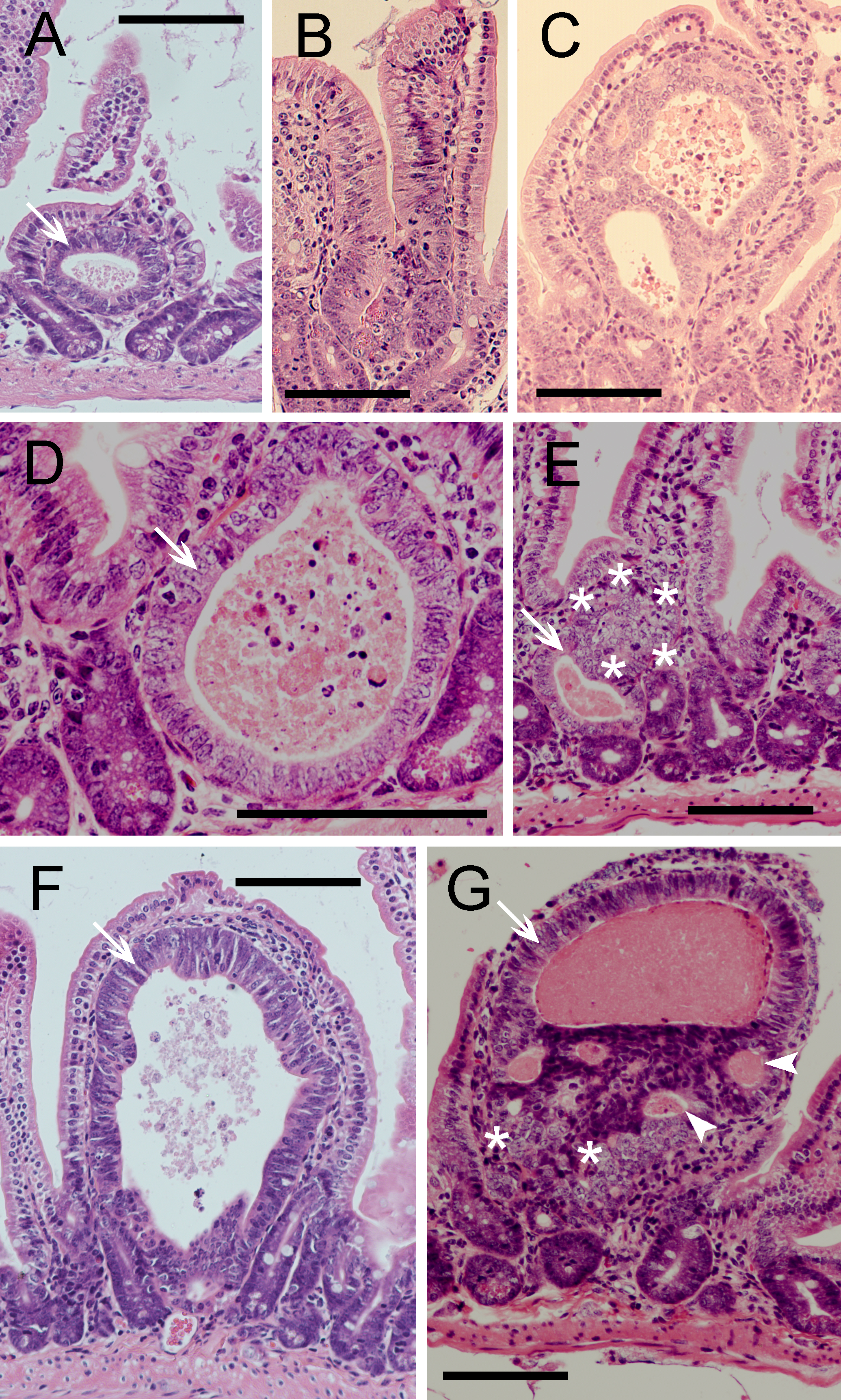
**

**Fig. S1**


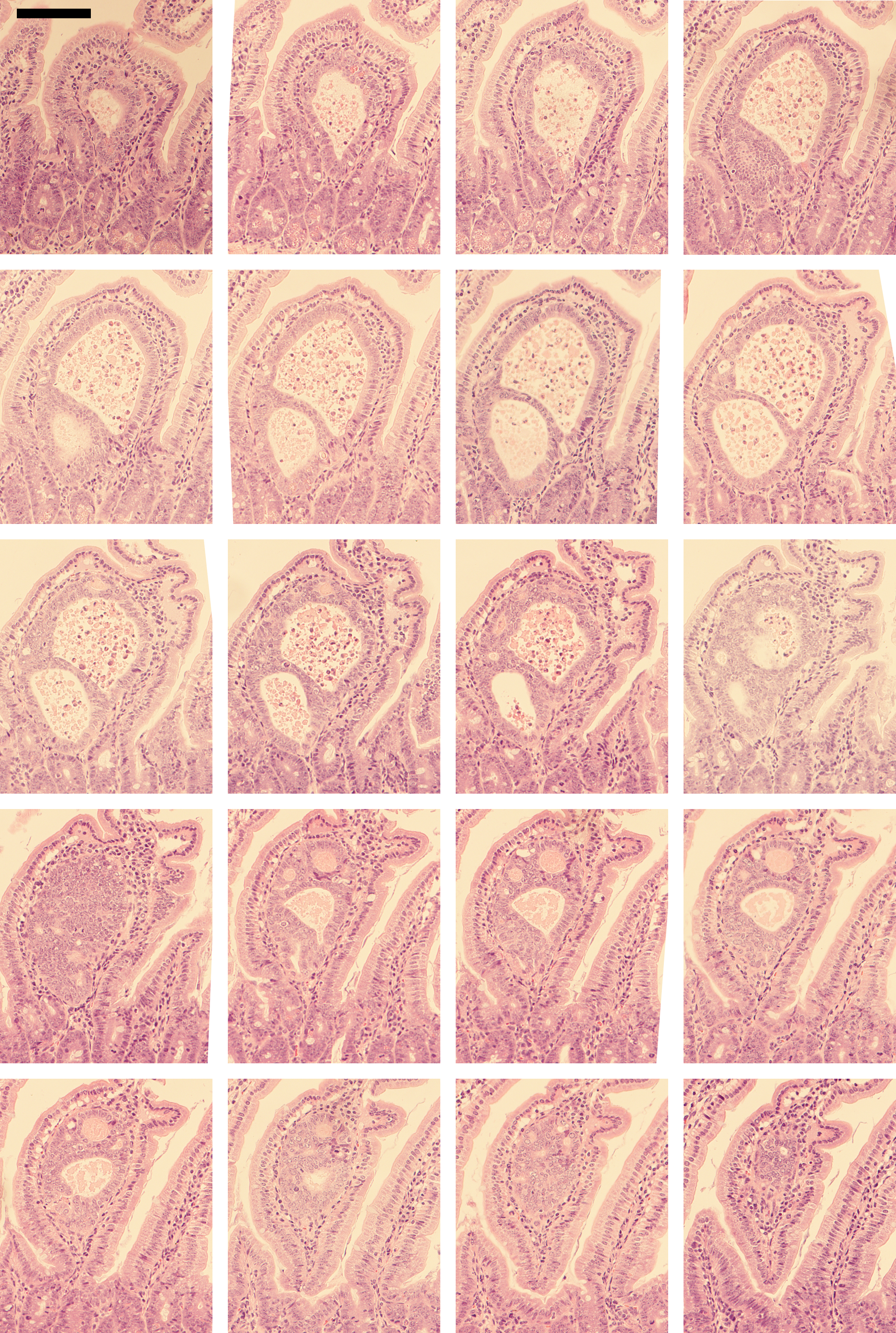


**Fig. S2**


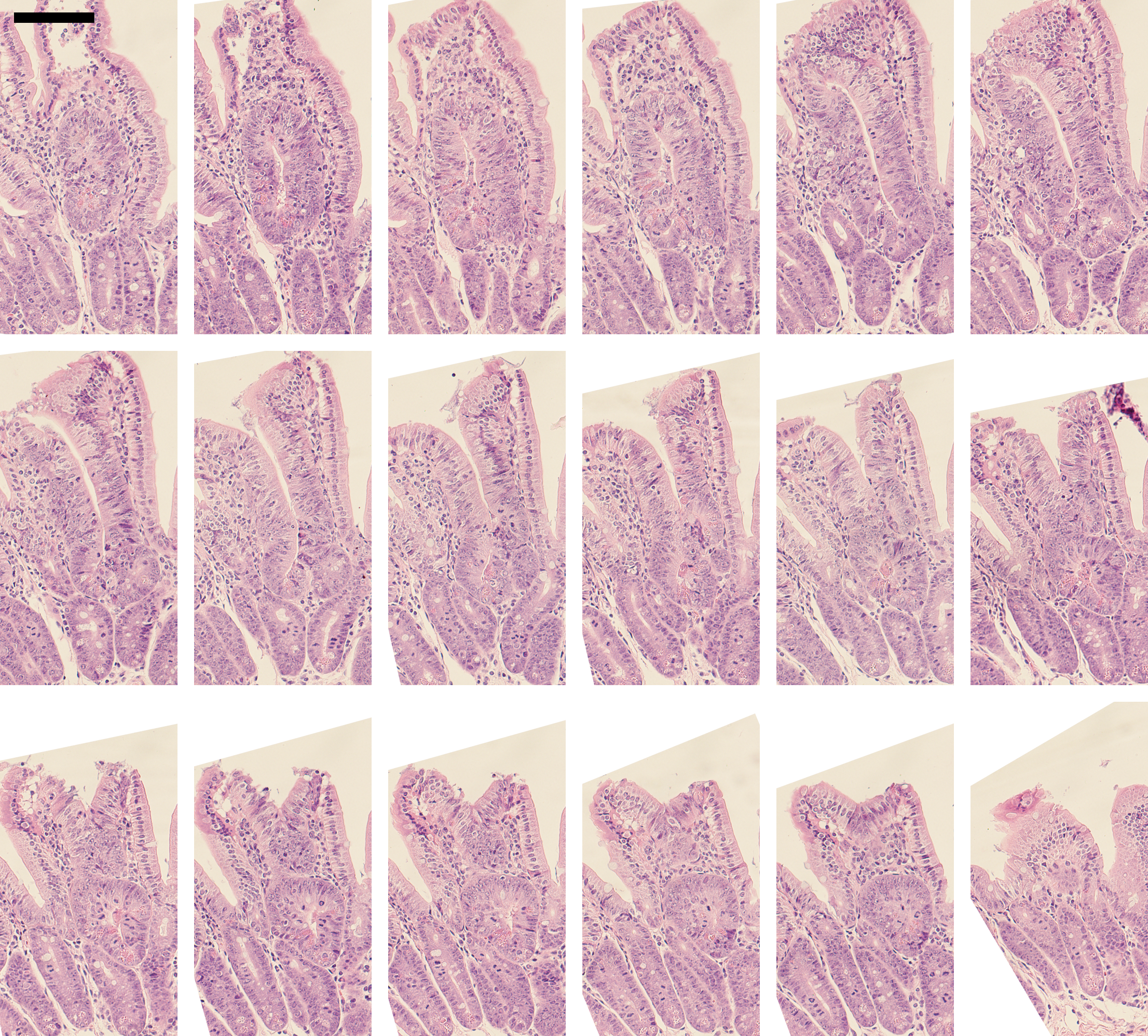


**Fig. S3**

**
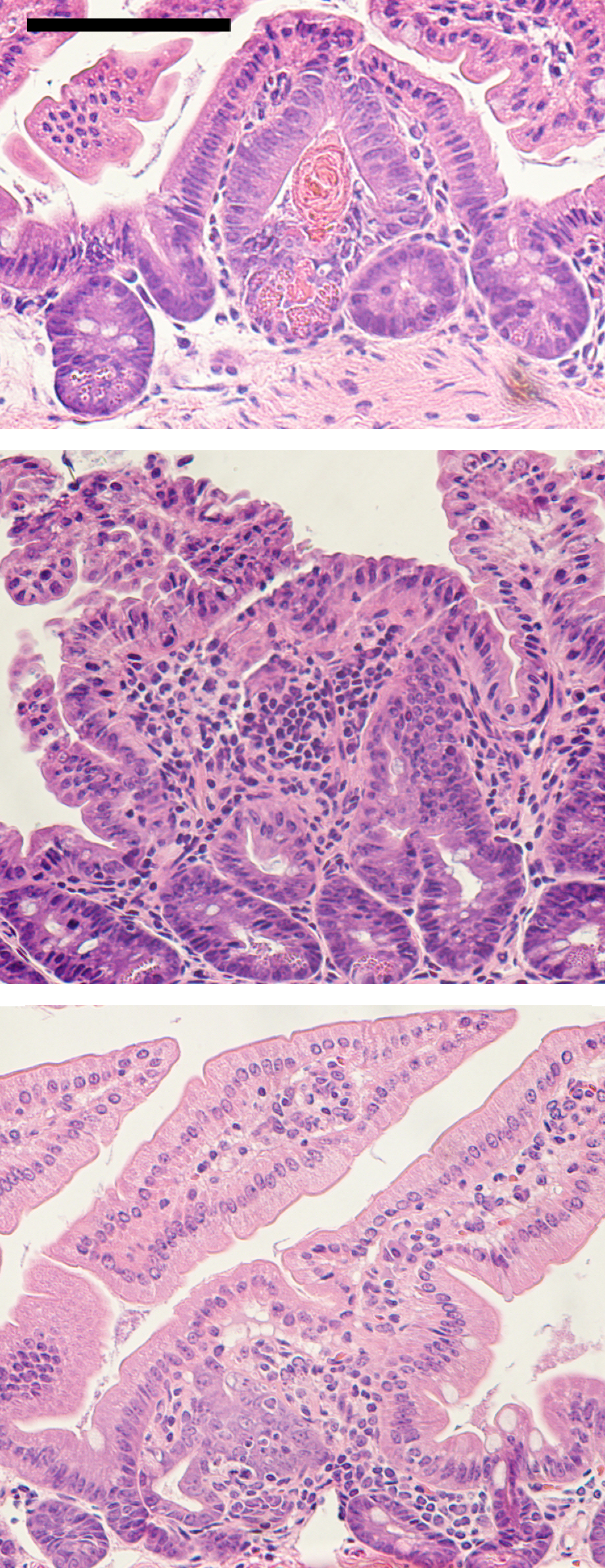
**

**Fig. S4**


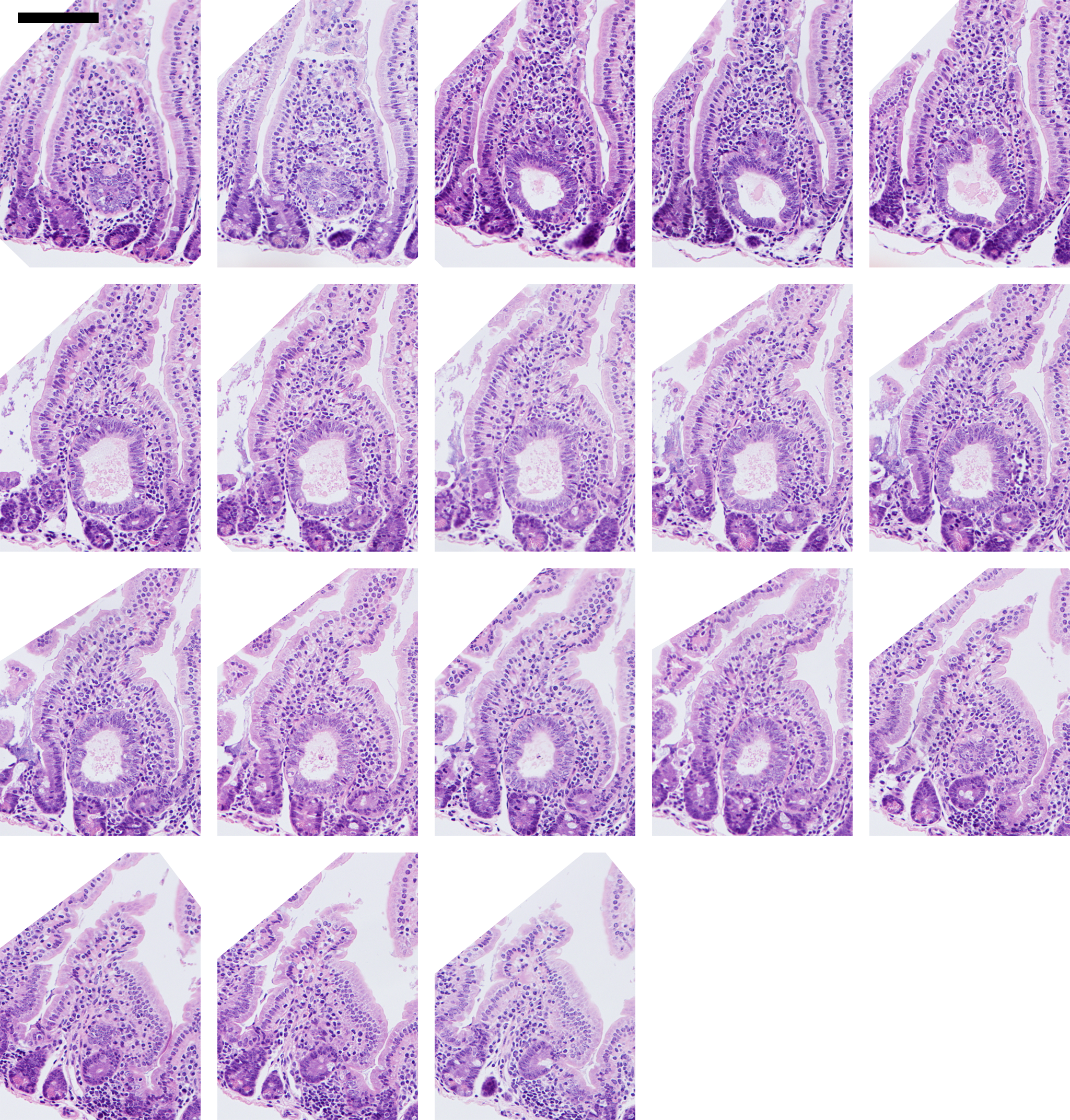


**Fig. S5**

**
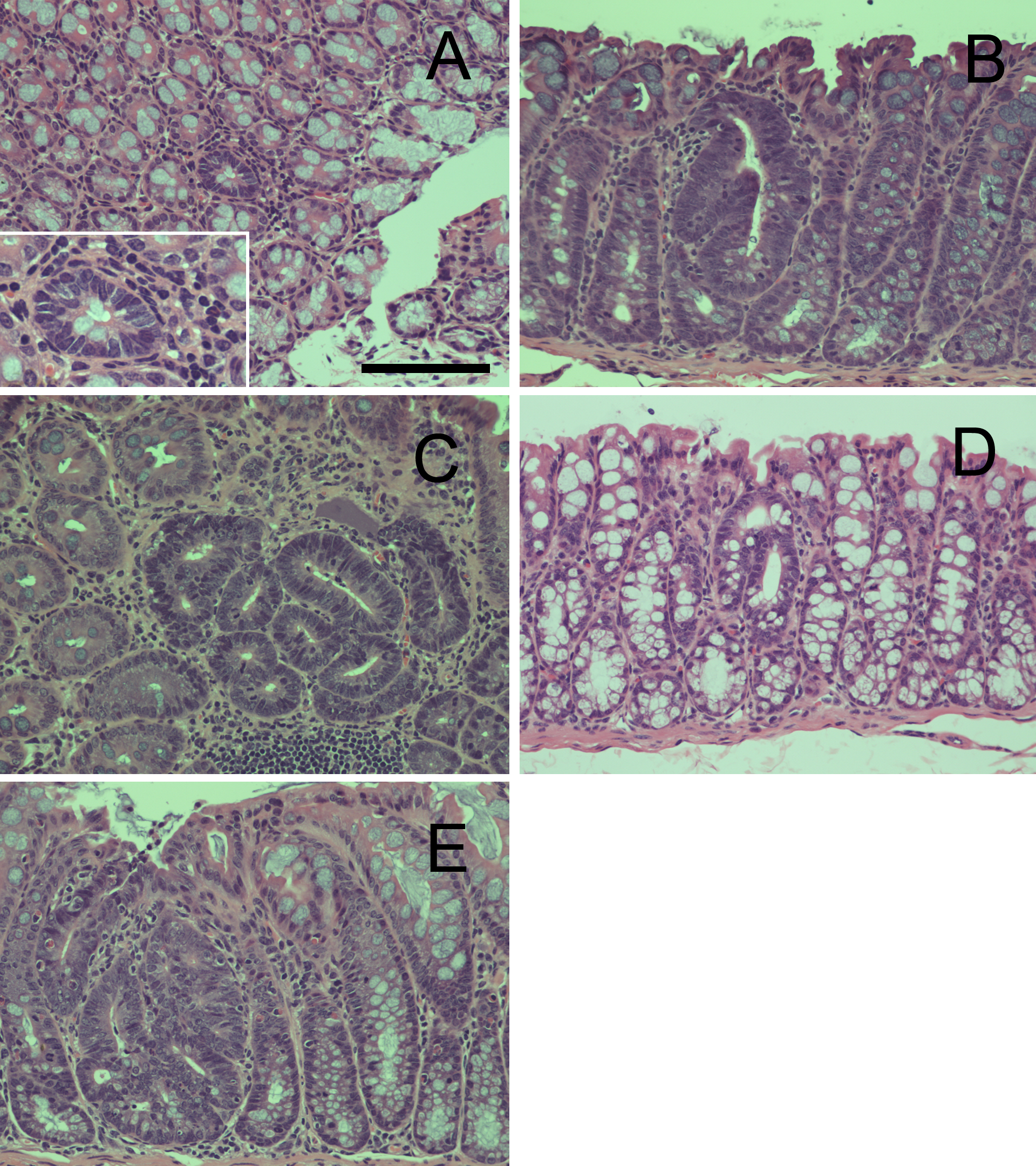
**

**Fig. S6**

**
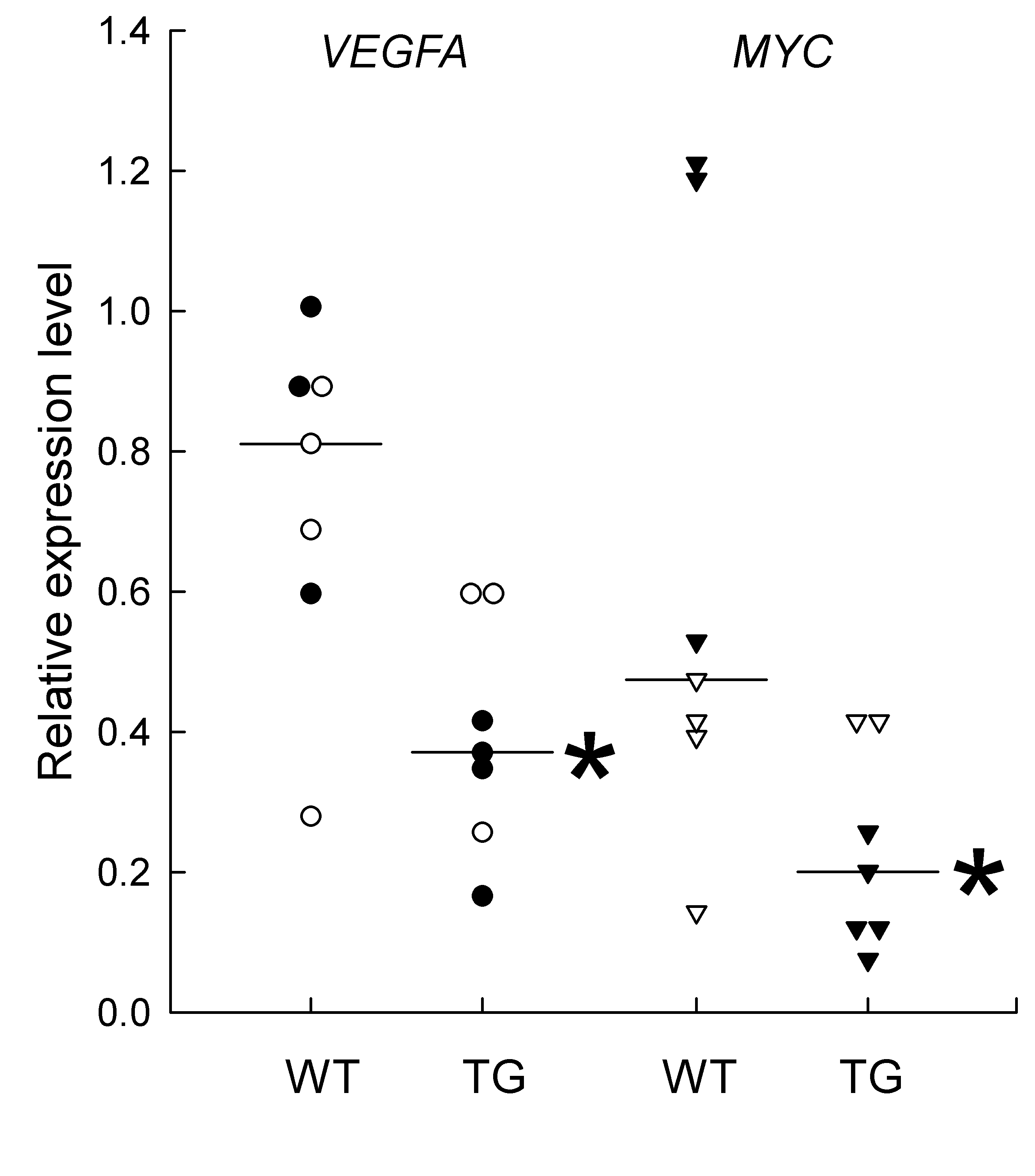
**

**Fig. S7**


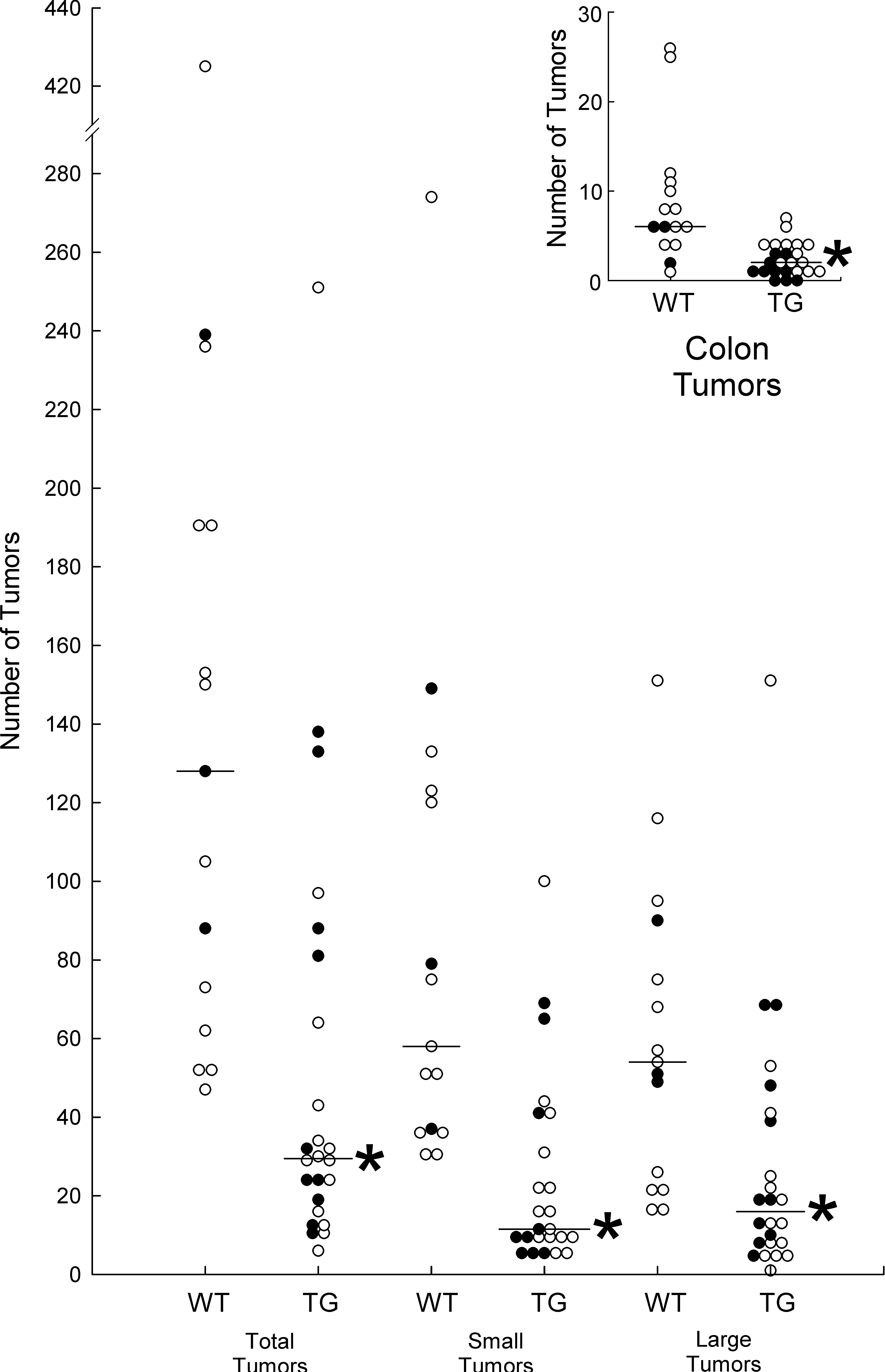


**Fig. S8**

**
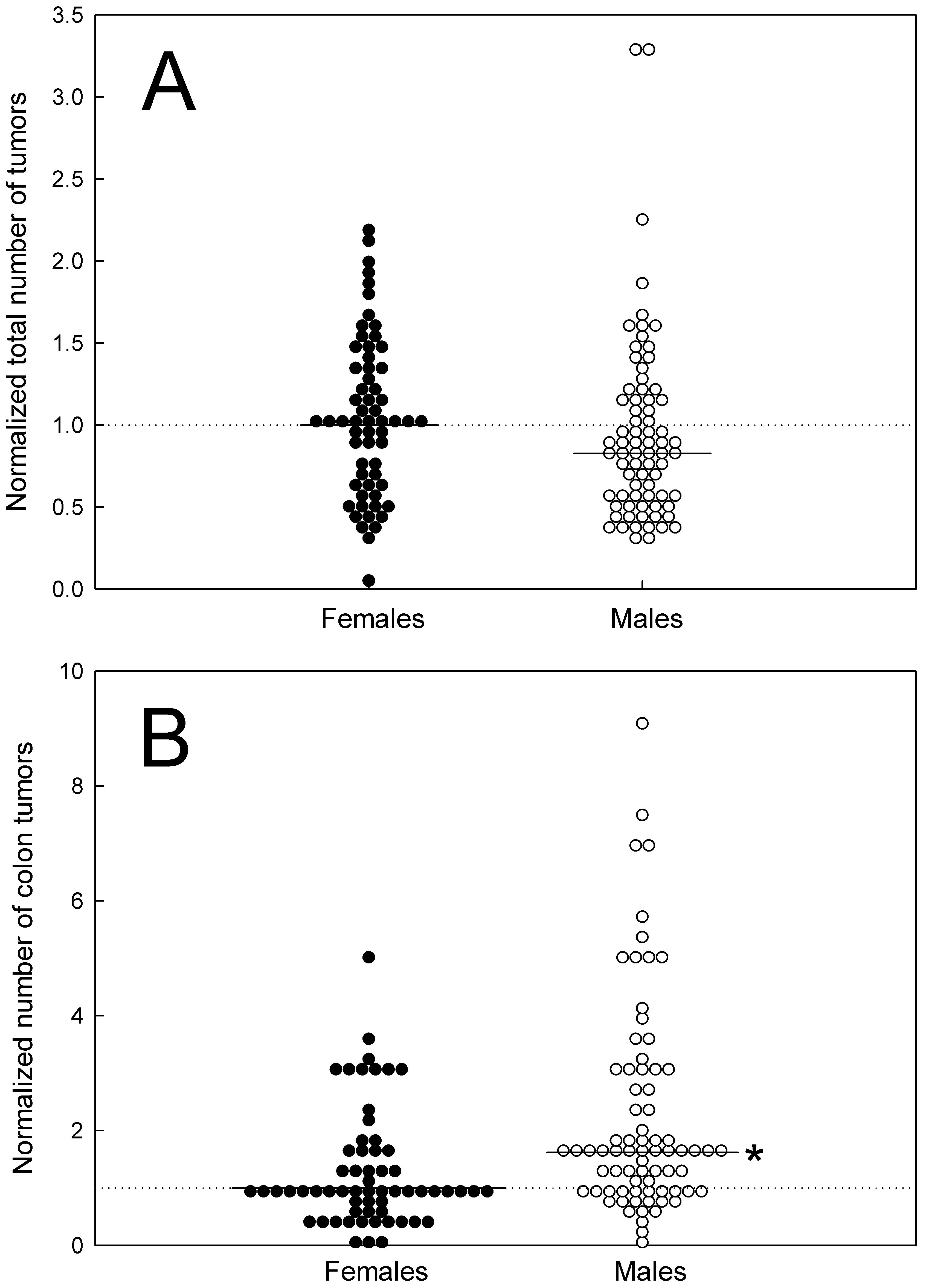
**

**Fig. S9**
